# FXR inhibition: an innovative prophylactic strategy against SARS-CoV-2 infection

**DOI:** 10.1038/s41392-023-01390-y

**Published:** 2023-03-21

**Authors:** Zhe Li, Shuai Wang, Fangfang Zhou

**Affiliations:** Institutes of Biology and Medical Science, Soochow University, Suzhou, 215123 China

**Keywords:** Infectious diseases, Drug regulation

A recent study by Brevini et al. was published in *Nature* and proposed a critical role of farnesoid X receptor (FXR) signaling in controlling ACE2 expression. Inhibitors of FXR signaling, ursodeoxycholic acid (UDCA) and z-guggulsterone (ZGG), have been proven to reduce ACE2 expression and susceptibility to SARS-CoV-2 infection in human tissues, providing an innovative strategy for SARS-CoV-2 treatment and prevention.^1^

Although the treatment of SARS-CoV-2 has improved with the development of therapeutic drugs, vaccines, and monoclonal antibodies, new SARS-CoV-2 variants continue to emerge, causing significant morbidity and mortality. These newly emerged variants evade the neutralizing effect of monoclonal antibodies and the protective effects of the vaccine.^2^

**Fig. 1:**
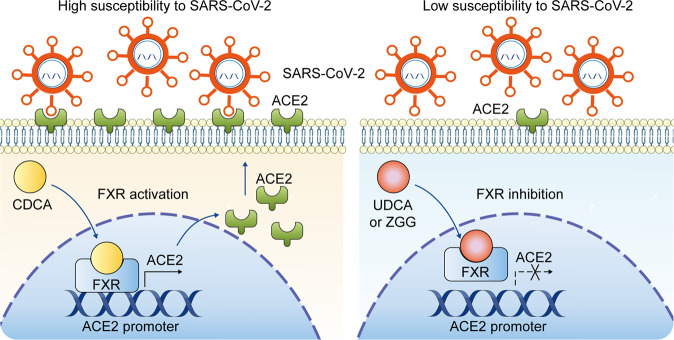
The schematic diagram of FXR signaling controlling ACE2 expression. Left, CDCA binds to and activates FXR, which further induces ACE2 expression. Right, FXR inhibitors, including UDCA and the ZGG, diminish ACE2 expression and thereby reduce the susceptibility to SARS-CoV-2 infection

Angiotensin-converting enzyme 2 (ACE2) acts as the main receptor of SARS-CoV-2. Brevini et al. found that the bile acid receptor, farnesoid X receptor (FXR), directly regulates ACE2 transcription in multiple tissues. To study the regulatory mechanism of ACE2 transcription, Brevini et al. selected gallbladder cholangiocyte organoids because of their high ACE2 expression, susceptibility to SARS-CoV-2 infection, and appropriate innate immune responses. Importantly, the absence of bile acids (CDCA) causes cholangiocyte organoids to lose ACE2 expression. This demonstrates that CDCA is required for ACE2 expression because it binds to and activates the transcription factor FXR.^1^ Brevini et al. hypothesized that CDCA controls ACE2 transcription via FXR (Fig. 1).

Knockdown of FXR by shRNA in cholangiocyte organoids prevented CDCA-induced ACE2 expression. Chromatin immunoprecipitation revealed that FXR directly binds to the ACE2 promoter. Suppression of FXR signaling by ZGG or UDCA disrupts the binding of FXR to the ACE2 promoter, thereby decreasing ACE2 expression. In addition, Brevini et al. found that the regulation of ACE2 expression by FXR also exists in organoids derived from the respiratory, biliary, and intestinal epithelium, indicating that FXR modulates ACE2 expression in multiple organs.^1^

Subsequently, researchers investigated whether inhibition of FXR signaling could decrease the susceptibility to SARS-CoV-2 in vitro. Suppression of FXR signaling with UDCA or ZGG reduced SARS-CoV-2 infection in gallbladder cholangiocytes, airways, and intestinal organoids. Accordingly, FXR depletion or ACE2 overexpression ablated the effect of UDCA or ZGG on viral infection, confirming that UDCA and ZGG reduce susceptibility to SARS-CoV-2 via FXR signaling (Fig. 1).

To validate these findings in vivo, the researchers treated FVB/N mice and Syrian Golden Hamsters with UDCA and found that pre-treatment with UDCA downregulated ACE2 expression and prevented SARS-CoV-2 transmission, demonstrating the prophylactic potential of UDCA against SARS-CoV-2.

Subsequently, Brevini et al. validated these observations using a pair of human lungs (declined for transplantation) maintained with ex situ normothermic perfusion (ESNP). Clinical doses of circulating UDCA downregulated ACE2 expression in the lung and significantly reduced SARS-CoV-2 infection. Then, the researchers repeated ESNP with human liver grafts, and found that UDCA treatment also downregulated ACE2 in the gallbladder cholangiocytes and circulating perfusate, and reduced SARS-CoV-2 infection ex vivo. To our knowledge, this is the first study to test the effect of a drug on an entire human organ.

To strengthen their study, the authors recruited 8 volunteers and treated them with UDCA at a standard therapeutic dosage of 15 mg/kg/day for 5 days. The results showed that UDCA reduced ACE2 levels in the nasal epithelium of humans. The authors further explored the effect of UDCA treatment on the outcome of SARS-CoV-2 patients. The prognosis of SARS-CoV-2 patients treated with UDCA is better than those without UDCA, including reduced hospitalization, reduced ICU admission, and reduced death. These results clearly suggest that UDCA can improve SARS-CoV-2 outcomes.^1^

SARS-CoV-2 entry depends on spike protein binding to ACE2 receptors, which serves as a reasonable therapeutic target. ACE2 is widely expressed in the respiratory systems, digestive tract, liver, kidney, heart, brain, and other organs, which exhibit strong spatiotemporal heterogeneity in COVID-19 pathophysiology. Despite the moderate ACE2 expression level in lungs, pneumonia is the primary symptom of COVID-19 patients, presumably because respiratory tract is the main transmission route.^3^ Accumulating evidence implicates the ACE2 expression in patients is positively related to the susceptibility to SARS-CoV-2, severity, and mortality. Innovatively, the results of Brevini et al. revealed that inhibiting FXR signaling with UDCA or ZGG inhibits ACE2 expression, thereby reducing the susceptibility to SARS-CoV-2 infection. To date, several vaccines and monoclonal antibodies have been developed to neutralize spike proteins. However, the newly emerged variants usually harbor mutations in the spike protein and are not recognized by the vaccine response monoclonal antibodies. The results of Brevini et al. provide a novel approach to impede viral entry by downregulating the ACE2, which has an advantage over strategies targeting the spike protein. Since ACE2 is an endogenous host protein, the inhibitory effect of UDCA on ACE2 transcription is probably unaffected by mutations in the virus spike proteins. Therefore, UDCA treatment may prevent the entry of various SARS-CoV-2 variants and other viruses using the same receptor. The authors validated the prophylactic effects of UDCA against SARS-CoV-2 in organoids, mice, hamsters, and human organ models. Moreover, UDCA was proven to improve the outcome of SARS-CoV-2 patients. Although UDCA is approved for treatment of cholangiopathy disease, it has minimal side effects in some patients, including hepatitis, cholangitis, diarrhea, mutagenic effects, etc.^4^ ZGG belongs to steroid drug which has common toxicity profile including immunosuppression, liver and gastrointestinal disorders, weight gain, etc. Thus, the authors did not support the use of UDCA or ZGG for SARS-CoV-2 treatment until robust clinical evidence was available.

Apart UDCA and ZGG, several compounds have been documented to reduce ACE2 expression. Given that ACE2 is an IFN-stimulated gene, inhaled corticosteroids and fluticasone propionate are proven to inhibit ACE2 and reduce susceptibility to SARS-CoV-2 via suppressing innate immune signaling.^5^ Interleukin-13 reduces ACE2 expression but increases the expression of transmembrane protease serine 2 (TMPRSS2), which promotes the fusion of SARS-CoV-2 and cellular membranes. While other FXR antagonists, such as hyocholic acid, tauro-β-muricholic acid, AGN34, tuberatolides, atractylenolides, andrographolides, GW4064 derivatives and 1,3,4-trisubstitutedpyrazolones, may reduce ACE2 expression and have the potential to be developed into powerful drugs for SARS-CoV-2 treatment.^6^

In conclusion, the approved drug UDCA and over-the-counter ZGG have significant potential as preventive and therapeutic drugs against SARS-CoV-2 infection and are less prone to viral resistance. On the basis of these findings, it will be of broad application prospects and great significance to develop new drugs or therapeutic strategies with minimal side effects for SARS-CoV-2 treatment and prevention.
